# Seasonal Dynamics of the Volatile Metabolome and Aroma Contribution in Xinyang Maojian Green Tea

**DOI:** 10.3390/biology15120925

**Published:** 2026-06-13

**Authors:** Jie Zhou, Yiwei Yang, Zhijie Wei, Yu Che, Jilai Cui

**Affiliations:** 1 Dabie Mountain Laboratory, College of Tea and Food Science, Xinyang Normal University, Xinyang 464000, China; zhoujie@xynu.edu.cn (J.Z.);; 2 Henan Key Laboratory of Tea Plant Biology, College of Tea and Food Science, Xinyang Normal University, Xinyang 464000, China; 3 Henan International Joint Laboratory of Tea-oil Tree Biology and High-Value Utilization, College of Tea and Food Science, Xinyang Normal University, Xinyang 464000, China

**Keywords:** Xinyang Maojian (XYMJ), volatile components, seasonal dynamics, aroma contribution

## Abstract

Seasonal variation in aroma quality is critical for commercial grading of Xinyang Maojian (XYMJ) green tea, and how seasonal changes shape its volatile composition and aroma profile remains unclear. This study investigated the volatile profiles of XYMJ harvested in spring, summer, and autumn using headspace solid-phase microextraction-gas chromatography-mass spectrometry (HS-SPME-GC-MS) and odor activity value (OAV) analysis. A total of 93 volatile compounds were identified, with alkenes, alcohols, and esters being the most numerous chemical classes. Total volatile content decreased significantly seasonally (*p* < 0.05), being highest in spring (1716.68 μg/kg), followed by summer (1566.72 μg/kg) and autumn (1378.21 μg/kg). PCA and PLS-DA clearly distinguished seasons. Using a dual-filtering strategy (variable importance in the projection > 1.0 and *p* < 0.01), 14 differential volatile metabolites were identified as core seasonal markers. Geraniol, *cis*-jasmone, and indole were identified as key drivers of the premium floral fragrance in spring XYMJ, while *cis*-3-hexenyl hexanoate and linalool peaked in the summer harvest. OAV results and cross-modal sensory interaction principles suggest that the superior flavor of spring XYMJ arises from both higher aromatic intensity and an optimized aroma-taste balance. These findings provide useful insights into the seasonal variations in the metabolic and chemical profiles of XYMJ, enhancing our understanding of the biochemical markers associated with its production timeline.

## Simple Summary

Xinyang Maojian is a famous Chinese green tea, and its flavor quality changes greatly with harvest season, which affects consumer preference and market value. However, it remains unclear how seasonal changes shape its aroma. This study aimed to explore seasonal differences in aroma compounds of Xinyang Maojian and identify key substances driving flavor variations. We analyzed tea samples from spring, summer and autumn, measured aroma-related chemicals and evaluated their flavor contributions. Results showed spring tea had the richest floral aroma, summer tea had stronger fruity notes, while autumn tea had weaker overall fragrance. These findings help guide seasonal tea grading, optimize cultivation and processing, and support sustainable development of the local tea industry, benefiting both farmers and consumers.

## 1. Introduction

Tea (*Camellia sinensis* (L.) Kuntze) is one of the most widely consumed non-alcoholic beverages worldwide, valued for its distinctive flavor and numerous health-promoting bioactive compounds [1,2,3]. Among various sensory attributes, aroma is widely recognized as a key determinant of tea quality and consumer preference [4]. The complex aroma of tea originates from a mixture of hundreds of volatile compounds, and the precise composition and relative abundance of these volatiles together define the characteristic flavor fingerprint of a specific tea type [5].

Xinyang Maojian (XYMJ) is celebrated as one of China’s top ten famous green teas, produced in the Xinyang region of Henan Province—the northernmost traditional tea-producing area in China [6]. With a tea-growing history spanning over 2000 years, XYMJ has long been renowned for its delicate appearance, bright green liquor, brisk taste, and notably high, long-lasting floral aroma [7]. As a premium green tea, XYMJ holds significant economic importance, supporting local farmers, driving regional agricultural development, and maintaining a strong market reputation for high quality. Understanding the chemical basis of its aroma and how it varies with environmental factors is thus of both scientific and practical importance for quality evaluation and improvement.

The aroma and taste quality of tea are highly sensitive to environmental and ecological conditions, including temperature [8], light wavelength [9], and weather condition [10], which fluctuate significantly with seasonal changes [11]. Previous studies have revealed that the high aroma of XYMJ is mainly attributed to terpenoids and fatty acid-derived volatiles. Compounds such as linalool and its oxides, geraniol, *β*-ionone, nonanal, and *cis*-3-hexenyl hexanoate have been identified as key odorants with high odor activity values, contributing to its characteristic floral and tender notes [6,12]. However, seasonal variations in environmental stress often induce corresponding shifts in volatile metabolism. Typically, spring tea exhibits richer floral and fruity terpenoids and superior sensory quality, whereas summer and autumn teas tend to accumulate higher levels of green or grassy volatiles and phenolic compounds such as catechins, which are associated with harsher taste attributes [13,14,15].

In recent years, analytical techniques such as headspace solid-phase microextraction combined with gas chromatography–mass spectrometry (HS-SPME–GC–MS) have been widely employed in tea aroma research due to their high sensitivity and capability to comprehensively characterize volatile profiles [10,16]. This approach enables the detection and semi-quantitative analysis of a broad spectrum of aroma-active compounds with minimal sample preparation, providing a reliable foundation for elucidating tea flavor chemistry. At the same time, the introduction of chemometric and machine learning methods has greatly advanced the interpretation of complex aroma datasets. Conventional multivariate statistical analyses—such as principal component analysis (PCA), partial least squares discriminant analysis (PLS–DA), and hierarchical cluster analysis (HCA)—have been successfully applied to reveal intrinsic relationships among samples and to identify differential compounds among samples [17,18]. When used in combination, these analytical and statistical approaches provide complementary insights—allowing both visualization of global patterns and accurate identification of key differential volatile compounds that contribute to seasonal and sensory distinctions. However, most previous studies have focused only on a single harvest period or lacked systematic seasonal comparisons of the volatile metabolome. Consequently, the seasonal dynamics of aroma compounds in XYMJ, as well as the molecular basis driving its characteristic seasonal sensory discrepancies, remain poorly understood.

The present study aims to address this gap by systematically analyzing the seasonal variations in volatile compounds in Xinyang Maojian green teas harvested during spring, summer, and autumn. Using HS-SPME–GC–MS, the study aims to identify and quantify the complete volatile profiles across the three major harvesting seasons, and to elucidate the variation and transformation patterns of key aroma-active compound classes. The findings are expected to enhance the understanding of how seasonal environmental fluctuations affect the biosynthesis and accumulation of aroma compounds, providing insights for tea-quality regulation and sustainable production management.

## 2. Materials and Methods

### 2.1. Manufacture of XYMJ

XYMJ were produced according to the following procedures: Fresh tea shoots with one bud and two leaves were collected in Shihe District, Xinyang, Henan, China. Three independent biological replicates were set for each season, and each replicate was sampled from at least 30 randomly selected tea plants in separate plots to ensure representativeness. The plant material belonged to *Camellia sinensis* var. *sinensis* cv. Xinyang quntizhong, the primary landrace used for producing Xinyang Maojian green tea. XYMJ green tea was then manufactured using fresh tea leaves according to traditional processing steps, including fixing (140–160 °C, 3–5 min), rolling (room temperature, 7 min), shaping (90 °C, 20 min), and drying (110 °C for 10 min, followed by final baking at 60–70 °C for 30 min), by an experienced tea master in a local tea factory as previously described [6]. XYMJ green teas were categorized into three seasonal types based on their harvest dates: spring XYMJ (produced around the Qingming Festival, 4 April 2024), summer XYMJ (produced after the Guyu period, 19 April 2024), and autumn XYMJ (produced around the Bailu period, 17 September 2024). After collection, all tea samples were stored at −40 °C until analysis. In Chinese tea agronomy, Qingming and Guyu are pivotal solar terms defining leaf maturity. Although separated by only 15 days, leaves harvested after Guyu possess significantly higher maturity, leading to a profound discrepancy in XYMJ quality and justifying their separate classification.

### 2.2. Extraction of XYMJ Volatile Compounds with HS-SPME

To extract the volatiles of tea samples, an exact amount of 1.0 g of ground tea sample was weighed into a 60 mL extraction vial. Then, 10 μL of the internal standard (ethyl decanoate, 1 mg/L, 99%, Sigma-Aldrich, St. Louis, MO, USA) was added using a micropipette. According to the established method for tea volatile analysis [19], a stir bar was placed into the vial, followed by 30 mL of boiling water. The vial was immediately sealed and placed in a water bath maintained at 60 °C. After incubating for 10 min, the SPME fiber (DVB/CAR/PDMS, 50/30 μm, 1 cm length, Bellefonte, PA, USA) was inserted into the vial headspace and exposed for 50 min with continuous stirring at 300 rpm. Subsequently, the fiber was withdrawn and immediately inserted into the GC injection port for thermal desorption for 5 min. The HS-SPME extraction conditions were selected based on preliminary optimization to maximize the extraction efficiency of volatile compounds while avoiding thermal degradation or the formation of heat-induced artifacts, consistent with established protocols for green tea flavor analysis [6].

### 2.3. Analysis of XYMJ Volatile Compounds by GC/MS

The volatile analysis was performed on a 7890-7000D GC/MS (Agilent Technologies, Santa Clara, CA, USA) according to previous methods [6]. The injection of the SPME fiber was carried out in a splitless mode through the GC injection port at 250 °C. The volatiles were separated on a fused silica capillary column (DB-5MS, 30 m × 0.25 mm, 0.25 μm film thickness, J&W Scientific, Folsom, CA, USA). The separation was achieved using a temperature program as follows: 60 °C (hold 2 min), then increased to 150 °C at 3 °C/min (hold 2 min), and finally raised to 280 °C at 7 °C/min (hold 2 min). High-purity helium (>99.999%, Henan Yuanzheng Special Gas Co., Ltd., Xinxiang, China) was used as the carrier gas at a flow rate of 1 mL/min. The ionization voltage was 70 eV and the ion source temperature was 230 °C. Full scan mode was used and the mass range was m/z 35–350.

### 2.4. Identification and Quantitation of Volatile Compounds

To identify compounds detected by GC/MS, the retention index (RI) of each volatile was calculated and a mixture of *n*-alkanes (C8–C25) was injected under the same GC-MS conditions. In addition, the volatiles were also identified by comparing the mass spectra to those in the National Institute of Standards and Technology (NIST21) database. Compounds with a mass spectra similarity higher than 800 and an RI difference of <20 were selected as volatiles in samples [6]. Ethyl decanoate was used as the internal standard to quantify each volatile by comparing the peak area of each compound to that of ethyl decanoate using their respective characteristic ions (Table 1). Three independent biological replicates were performed for each group of tea samples, and the results were expressed as mean ± standard deviation.

### 2.5. OAV Calculation

The calculation of OAV is widely used in the screening of the contribution degree of aroma substances [21,22], and it was calculated as the ratio of its concentration to its corresponding odor threshold in water. All odor thresholds used in this study were determined in a water matrix from the literature references [6,20]. It is generally believed that when the OAV is greater than 1, the substances have a high contribution to aroma [23].

### 2.6. Statistical Analysis

The MassHunter Unknown Analysis software (MassHunter Workstation Unknown Analysis 10.1, Agilent) was used to analyze the raw data obtained from GC/MS. Prior to multivariate analysis, the volatile data were normalized to the peak area of the internal standard (ethyl decanoate) and further transformed using Pareto scaling to standardize the variance across variables. SPSS (v25) software was employed to analyze the differences between samples. Simca-14.1 was used for multivariate statistical analysis, including the unsupervised Principal Component Analysis (PCA) and the supervised Partial Least Squares Discriminant Analysis (PLS-DA). All data were statistically analyzed using Excel 2023, and bar charts were generated. TBtools-II (v2.371) was used to create heatmaps. Stacked bar plot combined with Euclidean distance-based hierarchical cluster analysis was performed using the Metware Cloud, a free online platform for data analysis (https://cloud.metware.cn).

### 2.7. AI-Use

For language editing of the manuscript, Gemini 3.5 Flash generative AI was used to polish English grammar, sentence expression and academic wording; no AI was involved in experiment, data analysis and original content writing of this study.

## 3. Results and Discussion

### 3.1. Volatile Profiles in XYMJ from Different Seasons

**Figure 1:**
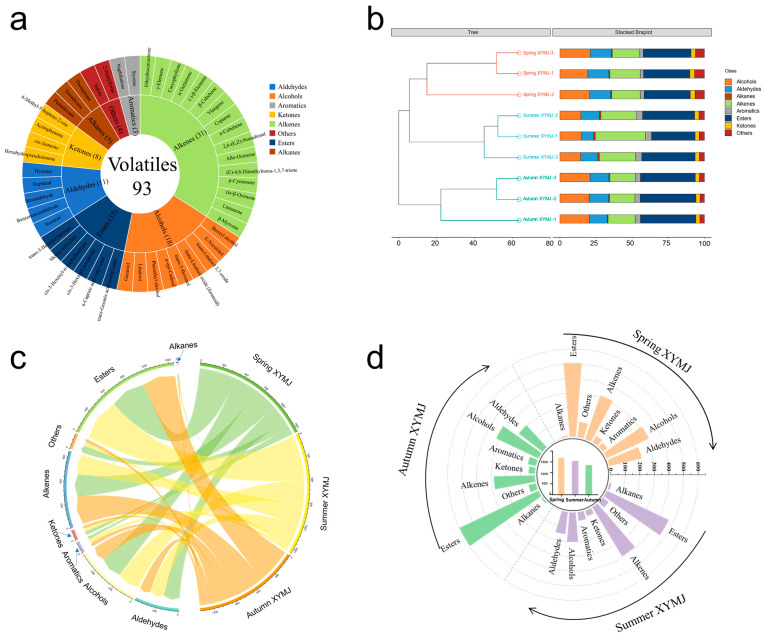
The volatile compounds in XYMJ tea across different seasons obtained from GC/MS. (**a**) Chemical classification and composition of volatile compounds identified in XYMJ; (**b**) hierarchical clustering analysis and stacked bar plot of volatile compound classes; (**c**) seasonal flow chart of volatile compound classes; (**d**) radial plot comparing the relative contents of major volatile classes across seasons.

The volatile components of XYMJ green tea produced across three different seasons were analyzed using HS-SPME-GC-MS, resulting in the identification of a total of 93 volatile compounds across all samples. A comparative analysis of the total relative volatile content revealed a clear seasonal trend: the content was highest in the spring XYMJ (1716.68 ± 28.41 μg/kg, DW), followed by the summer XYMJ (1566.72 ± 72.1 μg/kg), and the content in the autumn XYMJ (1378.21 ± 124.63 μg/kg) was significantly lower than that of the other two seasons (*p* < 0.05) (Table 2, Figure 1d).

These 93 volatile compounds were divided into nine chemical classes (Figure 1a). Alkenes, alcohols and esters were the most abundant groups in terms of compound number. In regard to total content, esters dominated across samples, with the highest level of 548.53 μg/kg in spring tea, followed by alkenes (349.02 μg/kg) and alcohols (344.06 μg/kg). Detailed numbers and percentage distributions of each class are listed in Table S1 (Supplementary Material). Collectively, these three classes contributed approximately 67% of the total volatile content in the spring sample. The formation of these major classes is mainly linked to key biosynthetic pathways: alcohols are generally derived from the hydrolysis of glycoside aroma precursors and the biosynthetic pathway of volatile terpenoid compounds [24], while esters are generated from the free fatty acids [25]. Notably, this distribution pattern aligns closely with findings from previous green tea studies. For instance, similar dominance of alkenes, alcohols, and esters has been reported in Longjing [23], Biluochun [26], and other premium Chinese green teas, where these three groups consistently account for over 60% of total volatile components. The prevalence of these compound classes across different green tea varieties highlights their conserved roles in shaping the characteristic fresh and floral aroma profiles of green tea.

### 3.2. Seasonal Dynamics of Volatile Compound Classes

The significant influence of season on the overall volatile profile was confirmed by hierarchical clustering analysis. The dendrogram in Figure 1b shows that samples clustered strongly according to the harvest seasons, confirming the distinct chemical separation driven by the harvest time.

The seasonal shifts in the major chemical classes are detailed in Table 2 and visualized by the flow chart (Figure 1c) and the radial plot (Figure 1d). Key dynamics in the class subtotals are as follows: Esters showed the highest content overall, peaking in summer XYMJ (597.85 μg/kg). The content in both spring XYMJ (548.53 μg/kg) and autumn XYMJ (467.69 μg/kg) was significantly lower than that in the summer one (*p* < 0.05). For alcohols, the total content was statistically similar between spring (344.06 μg/kg) and summer XYMJ (320.77 μg/kg) (*p* = 0.18), but the content in autumn XYMJ (202.42 μg/kg) was significantly lower than that of the other two seasons (*p* < 0.01). Aldehydes exhibited a clear decreasing trend from spring (249.95 μg/kg) to autumn (151.57 μg/kg) XYMJ, with content in summer and autumn XYMJ being significantly lower than that in the spring one. Alkenes displayed a fluctuating trend, decreasing from 349.02 μg/kg (spring XYMJ) to 281.49 μg/kg (summer), but then increasing sharply to the highest value of 392.06 μg/kg in autumn XYMJ. The accumulation of alkenes in autumn acts as an adaptive response for tea plants to resist abiotic and biotic stresses and shapes the unique aroma of autumn tea [27]. Aromatic compound contents in summer and autumn XYMJ were significantly higher than in spring. Conversely, alkane contents were highest in autumn compared to spring and summer. Ketones and heterocyclics showed a significant decline in content in summer and autumn XYMJ compared to the spring one (*p* < 0.05). The flow chart (Figure 1c) further highlights the substantial variations in esters and alkenes across the seasons, suggesting that they are major contributors to the overall chemical differences. These pronounced seasonal variations in major chemical classes will be further analyzed to identify the key odorants responsible for the seasonal divergence in XYMJ’s high aroma quality.

### 3.3. Differential Volatile Compound Analysis

**Figure 2:**
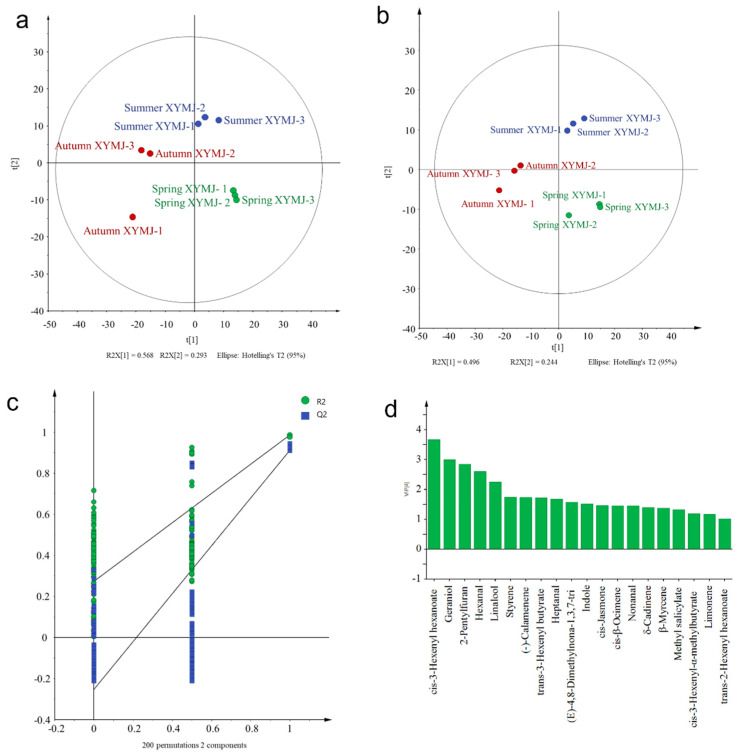
Analysis of the differential volatile compounds in XYMJ. (**a**) Scores plots of PCA (R^2^X = 0.978, Q^2^ = 0.938), (**b**) scores plots of PLS-DA (R^2^X = 0.988, R^2^Y = 0.999, Q^2^ = 0.995), (**c**) permutation test plot (200 times) of the PLS-DA model (R^2^ =0.275, Q^2^ = −0.251), (**d**) volatile components with the variable importance in the projection (VIP) higher than 1.

To characterize the seasonal divergence in volatile profiles of Xinyang Maojian (XYMJ) tea and to elucidate the underlying metabolic variations, chemometric analyses were conducted. An unsupervised Principal Component Analysis (PCA) was first applied to investigate the inherent structure of the dataset. As illustrated in the PCA score plot (Figure 2a), samples from the three harvest seasons—spring, summer, and autumn—were clearly and completely separated, with tight and non-overlapping clusters observed within each seasonal group. The first two principal components explained 56.8% (PC1) and 29.3% (PC2) of the total variance, respectively, resulting in a high cumulative contribution rate of 86.1%. These results strongly suggest that harvest season is the dominant factor driving the global variation in volatile metabolites of XYMJ tea throughout the production year.

To further identify the key volatile compounds contributing to the observed seasonal discrimination, a supervised Partial Least Squares Discriminant Analysis (PLS-DA) model was subsequently established (Figure 2b). The model demonstrated strong explanatory and predictive performance, with high goodness-of-fit parameters of R^2^X = 0.988 and R^2^Y = 0.999, and a cumulative cross-validated predictability (Q2) of 0.995, indicating that the variables effectively described the seasonal classification. To mitigate and evaluate the inherent risk of overfitting associated with a high variable-to-sample ratio, model robustness was rigorously assessed via both 7-fold cross-validation and a 200-iteration permutation test (Figure 2c). The resulting Q^2^ intercept value of −0.251, together with consistently lower permuted R^2^ and Q^2^ values compared with those of the original model, confirmed the absence of overfitting. Collectively, these validation results demonstrate that the PLS-DA model was statistically reliable and suitable for the subsequent screening of season-specific volatile biomarkers.

The contribution of volatile compounds to seasonal discrimination was evaluated using the variable importance in the projection (VIP) metric, where values > 1.0 indicate significant contributors. Among the 20 substances that met this criterion (Figure 2d), a strict dual-filtering strategy applying an additional threshold of *p* < 0.01 (ANOVA) ultimately prioritized 14 differential volatile metabolites as the core chemical markers driving the seasonal aroma shifts.

**Figure 3:**
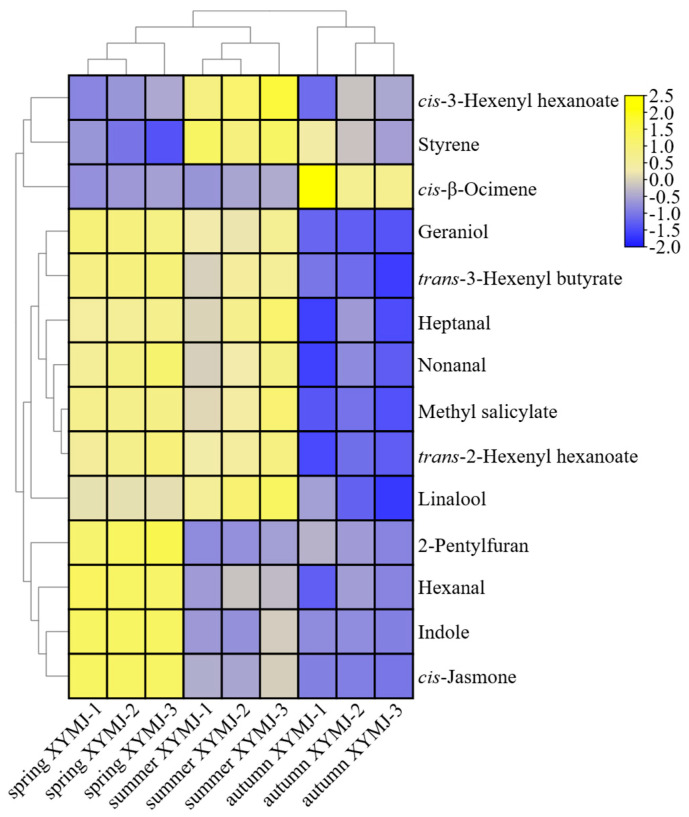
Hierarchical clustering heat map of volatiles in XYMJ tea with VIP > 1. Yellow color indicates the higher content in XYMJ, while blue color indicates low content. The dendrograms represent the hierarchical clustering of both volatile metabolites (**rows**) and tea samples (**columns**) based on Euclidean distance.

The seasonal accumulation patterns of the 14 identified volatile markers revealed distinct aroma profiles among spring, summer, and autumn XYMJ samples (Figure 3). Spring-harvested XYMJ was characterized by significantly elevated levels of premium floral-associated volatiles, notably geraniol (VIP = 2.98) and *cis*-jasmone. This metabolic profile is consistent with previous reports demonstrating that relatively low temperatures during early spring can stimulate the methylerythritol phosphate (MEP) pathway [28], thereby enhancing monoterpene biosynthesis. As a result, floral monoterpenes such as geraniol preferentially accumulate, contributing to the elegant and desirable aroma quality typical of high-grade spring teas. In contrast, summer XYMJ exhibited a pronounced enrichment of *cis*-3-hexenyl hexanoate, which displayed the highest VIP value among all discriminant markers (VIP = 3.65), accompanied by increased levels of linalool and styrene. *Cis*-3-hexenyl hexanoate is a representative green leaf volatile (GLV) ester derived from fatty acid oxidation via the lipoxygenase (LOX) pathway, in which alcohol dehydrogenase (ADH) mediates the conversion of C6 aldehydes to alcohols and their subsequent esterification. The accumulation of such compounds is closely associated with increased LOX and ADH activities under abiotic stress conditions, particularly elevated temperature and light exposure [29,30,31]. Autumn XYMJ exhibited the lowest concentrations for most differential volatiles. This trend is consistent with the overall reduction in total volatile intensity observed in the comprehensive profiling, and may reflect a seasonal decline in aroma-related metabolic activity as tea plants enter the late growth stage, during which both volatile composition and precursor metabolism are significantly affected by harvest season [32,33].

In summary, the combined chemometric approach successfully identified 14 key volatile markers that define the seasonal identity of XYMJ. The dynamic variations in these markers not only provide a molecular basis for the superior aroma quality observed in spring harvests but also reflect the tea plant’s complex biochemical adaptation to seasonal environmental stress.

### 3.4. Evaluation of Differential Aroma Contribution via OAV

To further evaluate the individual contribution of volatile compounds to the characteristic aroma of Xinyang Maojian (XYMJ), the Odor Activity Value (OAV) was calculated as a key metric. Generally, volatile compounds with an OAV > 1 are considered to contribute to the overall aroma profile, while those with an OAV > 10 are classified as major contributors [34]. Despite the identification of 93 volatile substances, the OAV results demonstrate that the typical fragrance of XYMJ is governed by a relatively small subset of highly active odorants, confirming that the key odorant concept is central to its quality (Table 3). Among these, linalool (floral/lavender) and nonanal (fatty/citrus) were identified as the aroma backbone of XYMJ, maintaining high active values regardless of the harvest season. Linalool, in particular, displayed OAVs ranging from 405 to 564, identifying it as the foundational fragrance driver of XYMJ. As noted in the established literature, linalool and its oxides are ubiquitous markers for high-quality green tea due to their exceptionally low odor thresholds and pleasant floral profiles [1]. Furthermore, the persistent high OAVs of nonanal and heptanal contribute a refreshing citrus-like undertone, which is a hallmark feature of premium Chinese green teas [5].

The superior aroma quality of spring XYMJ is primarily driven by a specific synergy of premium floral markers, notably geraniol (OAV = 20) and *cis*-jasmone (OAV = 4), which reached their peak concentrations in the spring samples. This aligns with previous studies on XYMJ confirming that spring harvests possess higher OAVs for geraniol, which correlates significantly with superior sensory scores for freshness [7]. Interestingly, indole reached its odor threshold (OAV ≥ 1) only in the spring; at such low concentrations, indole acts as a fragrance fixative, effectively enhancing the naturalness and depth of the floral bouquet [1].

In contrast, summer XYMJ exhibited a perceptible shift toward fruity intensity, primarily attributed to the peak in *cis*-3-hexenyl hexanoate (OAV = 17.22). However, the most striking finding in the autumn group was the comprehensive decline in OAVs for nearly all floral odorants; for instance, geraniol dropped to 6.54, and *cis*-jasmone barely reached its threshold, providing a chemical explanation for the diminished aroma intensity in late-season harvests.

Beyond direct olfaction, these volatile shifts significantly modulate overall flavor perception through cross-modal sensory interactions. It is well established that flavor perception arises from the centralized integration of gustatory and olfactory inputs in the brain [35,36]. In the case of XYMJ, the significantly higher OAVs of floral and sweet-noted volatiles in the spring—such as geraniol, linalool, and *cis*-jasmone—may exert an aroma-induced suppression of bitterness. Pleasant floral and sweet odorants can induce a sweetness expectancy, thereby elevating the perceptual threshold for bitterness and astringency through cognitive modulation [37].

The decline of these aromatic “masking agents” in autumn XYMJ leads to a collapse of this sensory harmony. As the OAVs of floral markers like geraniol drop, the underlying bitter and astringent non-volatile compounds (such as catechins and caffeine) become more perceptible to the palate [38]. This explains why autumn tea is frequently perceived as bitter and astringent, besides higher catechins in autumn tea [39]. Consequently, the excellence of spring XYMJ is a result of both its high volatile intensity and the optimized aroma–taste balance achieved through these complex cross-modal interactions.

In summary, the seasonal variations in the aroma profiles of XYMJ green tea were comprehensively characterized using HS-SPME-GC-MS coupled with OAV analysis. Alkenes, alcohols, and esters constituted the most dominant chemical classes out of the 93 identified volatiles, with the total content exhibiting a significant seasonal decline (Spring > Summer > Autumn). Multivariate statistical analysis successfully prioritized 14 differential metabolites as core chemical markers driving seasonal discrimination. Specifically, geraniol, *cis*-jasmone, and indole were identified as key markers for the superior floral aroma of spring XYMJ, whereas *cis*-3-hexenyl hexanoate and linalool peaked in summer, contributing to a more intense fruity profile. In contrast, autumn tea was characterized by a comprehensive reduction in these high-impact odorants, providing a molecular basis for its diminished aromatic intensity.

## 4. Conclusions

In conclusion, this study clarified the seasonal aroma variations in XYMJ green tea by combining HS-SPME-GC-MS, OAV analysis, and statistical filtering (PLS-DA and ANOVA). This practical approach provided a reliable way to pinpoint the core volatile markers responsible for seasonal differences. Our analysis suggests that the superior flavor of spring tea is driven not only by higher volatile concentrations but also by a better aroma–taste balance, where rich floral volatiles may help suppress bitterness and astringency through cross-modal sensory interactions. From an industrial standpoint, these findings offer clear chemical benchmarks for the quality control and seasonal grading of XYMJ green tea. This knowledge can practically help local producers adjust processing and blending methods to improve the quality and market value of summer and autumn tea harvests.

## Figures and Tables

**Table 1:** Identification of volatile compounds in XYMJ and their odor thresholds.

No.	Name	CAS Registry Number ^a^	Chemical Class	Quantitation Ion	Confirmation Ions	RT ^b^	RI-1 ^c^	RI-2 ^d^	OT ^e^
1	Hexanal	66-25-1	Aldehydes	64	64, 44, 41	3.19	800	800	5
2	*trans*-3-Hexenol	928-97-2	Alcohols	67	67, 82, 55	4.04	852	848	110
3	Styrene	100-42-5	Aromatics	104	104, 103, 79	4.9	893	897	65
4	2-Heptanol	543-49-7	Alcohols	45	45, 83, 55	4.95	900	900	65
5	Heptanal	111-71-7	Aldehydes	70	70, 55, 44	5.2	901	908	2.8
6	Benzaldehyde	100-52-7	Aldehydes	106	106, 105, 77	6.8	962	963	350
7	6-Methyl-5-hepten-2-one	110-93-0	Ketones	108	108, 69, 55	7.43	986	984	160
8	2-Pentylfuran	3777-69-3	Others	72	72, 138, 82	7.56	993	995	5.8
9	β-Myrcene	123-35-3	Alkenes	93	93, 69, 41	7.7	991	993	15
10	Limonene	138-86-3	Alkenes	68	68, 93, 67	8.9	1030	1029	200
11	Benzyl alcohol	100-51-6	Alcohols	108	108, 107, 79	8.99	1036	1031	2546
12	Benzeneacetaldehyde	122-78-1	Aldehydes	76	76, 120, 92	9.33	1045	1042	6.3
13	*cis*-β-Ocimene	3338-55-4	Alkenes	93	93, 79, 41	9.6	1038	1047	34
14	Acetophenone	98-86-2	Ketones	105	105, 77, 51	10.34	1065	1066	65
15	α-Phenylethanol	98-85-1	Alcohols	107	107, 122, 79	10.35	1061	1066	479
16	*cis*-Linalool oxide (furanoid)	5989-33-3	Alcohols	59	59, 94, 43	10.48	1074	1069	100
17	*trans*-Linalool oxide (furanoid)	34995-77-2	Alcohols	59	59, 94, 43	11.11	1086	1084	60
18	p-Cymenene	1195-32-0	Alkenes	117	117, 132, 91	11.34	1090	1088	85
19	Linalool	78-70-6	Alcohols	93	93, 121, 71	11.56	1099	1093	0.22
20	Nonanal	124-19-6	Aldehydes	84	84, 98, 56	11.77	1104	1105	1.1
21	Phenethyl alcohol	60-12-8	Alcohols	91	91, 122, 92	12.08	1116	1106	390
22	(*E*)-4,8-Dimethylnona-1,3,7-triene	19945-61-0	Alkenes	69	69, 82, 41	12.26	1116	1111	N.F.
23	Allo-Ocimene	673-84-7	Alkenes	121	121, 136, 105	12.82	1131	1126	N.F.
24	(*2E*,*6Z*)-Nonadienal	557-48-2	Aldehydes	88	88, 70, 41	13.78	1155	1154	N.F.
25	1-Nonanol	143-08-8	Alcohols	56	56, 70, 55	14.57	1173	1168	45
26	*trans*-Linalool oxide (pyranoid)	39028-58-5	Alcohols	68	68, 94, 59	14.6	1173	1168	3000
27	Naphthalene	91-20-3	Aromatics	128	128, 139, 127	14.87	1182	1174	450
28	*trans*-3-Hexenyl butyrate	16491-36-4	Esters	67	67, 82, 71	15.26	1187	1183	500
29	Methyl salicylate	119-36-8	Esters	120	120, 152, 92	15.46	1192	1187	40
30	Safranal	116-26-7	Aldehydes	107	107, 121, 91	15.7	1201	1192	N.F.
31	Decanal	112-31-2	Aldehydes	57	57, 55, 43	15.99	1206	1198	10,000
32	β-Cyclocitral	432-25-7	Aldehydes	137	137, 123, 109	16.6	1220	1212	3
33	Nerol	106-25-2	Alcohols	97	97, 93, 41	16.98	1228	1226	680
34	*cis*-3-Hexenyl-α-methylbutyrate	53398-85-9	Esters	67	67, 82, 57	17.2	1234	1226	N.F.
35	*cis*-3-Hexenyl isovalerate	35154-45-1	Esters	82	82, 67, 57	17.39	1238	1232	N.F.
36	Vinyl hexanoate	3050-69-9	Esters	99	99, 71, 43	17.63	1244	1238	N.F.
37	Geraniol	106-24-1	Alcohols	69	69, 68, 41	18.11	1255	1249	6.6
38	β-Homocyclocitral	472-66-2	Aldehydes	103	103, 107, 81	18.17	1254	1250	N.F.
39	Citral	1891-67-4	Aldehydes	104	104, 84, 41	18.8	1276	1263	N.F.
40	2-Methylnaphthalene	91-57-6	Aromatics	142	142, 141, 115	19.56	1298	1281	3
41	Indole	120-72-9	Others	117	117, 90, 89	19.63	1295	1283	40
42	Theaspirane	36431-72-8	Alkanes	138	138, 96, 82	20.64	1302	1304	N.F.
43	Methyl *trans*-geranoate	118-09-9	Esters	69	69, 114, 41	21.08	1324	1316	N.F.
44	*cis*-3-Hexenyltiglate	67883-79-8	Esters	82	82, 83, 67	21.12	1325	1317	N.F.
45	α-Cubebene	17699-14-8	Alkenes	105	105, 161, 119	22.09	1351	1342	N.F.
46	Dehydro-ar-ionene	30364-38-0	Aromatics	157	157, 172, 142	22.17	1354	1343	N.F.
47	Copaene	3856-25-2	Alkenes	161	161, 119, 105	23.17	1376	1366	N.F.
48	Ylangene	14912-44-8	Alkenes	119	119, 120, 105	23.17	1372	1366	N.F.
49	*cis*-3-Hexenyl hexanoate	31501-11-8	Esters	82	82, 99, 67	23.54	1380	1375	16
50	*n*-Hexyl hexanoate	6378-60-5	Esters	117	117, 99, 84	23.73	1384	1380	N.F.
51	β-Cubebene	13744-15-5	Alkenes	161	161, 105, 91	23.78	1389	1380	N.F.
52	(−)-β-Elemene	515-13-9	Alkenes	81	81, 93, 68	23.85	1391	1382	N.F.
53	*trans*-2-Hexenyl hexanoate	53398-86-0	Esters	99	99, 71, 43	23.88	1391	1383	781
54	*cis*-Jasmone	488-10-8	Ketones	164	164, 110, 79	24.16	1394	1389	7
55	Tetradecane	629-59-4	Alkanes	57	57, 71, 43	24.26	1400	1391	1000
56	Hexahydropseudoionone	1604-34-8	Ketones	58	58, 71, 42	24.46	1408	1395	N.F.
57	α-Gurjunene	489-40-7	Alkenes	204	204, 189, 161	24.55	1409	1398	N.F.
58	Caryophyllene	87-44-5	Alkenes	93	93, 133, 91	24.94	1419	1407	64
59	α-Ionone	127-41-3	Ketones	128	128, 136, 93	25.33	1426	1427	3.8
60	γ-Elemene	29873-99-2	Alkenes	121	121, 107, 93	25.56	1434	1423	N.F.
61	Dihydrocurcumene	1461-02-5	Alkenes	119	119, 204, 120	26.13	1448	1438	N.F.
62	Cadina-3,5-diene	267665-20-3	Alkenes	161	161, 119, 105	26.19	1458	1440	N.F.
63	α-Humulene	6753-98-0	Alkenes	93	93, 121, 80	26.31	1454	1444	N.F.
64	*trans*-Geranylacetone	3796-70-1	Ketones	43	43, 69, 41	26.4	1453	1447	60
65	(*E*)-*β*-Farnesene	18794-84-8	Alkenes	69	69, 133, 93	26.59	1457	1453	N.F.
66	*cis*-Muurola-4(15),5-diene	157477-72-0	Alkenes	161	161, 105, 91	26.7	1463	1456	N.F.
67	Cadina-1(6),4-diene	16279-00-3	Alkenes	161	161, 204, 105	27.16	1481	1473	N.F.
68	Dehydro-β-ionone	1203-08-3	Ketones	175	175, 131, 115	27.57	1485	1488	N.F.
69	*trans*-β-Ionone	79-77-6	Ketones	177	177, 178, 135	27.68	1486	1491	0.007
70	Bicyclosesquiphellandrene	54324-03-7	Alkenes	161	161, 105, 91	27.85	1489	1496	N.F.
71	Valencene	4630-07-3	Alkenes	161	161, 204, 105	28	1492	1501	N.F.
72	α-Muurolene	10208-80-7	Alkenes	105	105, 204, 161	28.24	1499	1509	N.F.
73	Pentadecane	629-62-9	Alkanes	57	57, 71, 43	28.29	1500	1511	13,000,000
74	Dibenzofuran	132-64-9	Others	168	168, 169, 139	28.52	1514	1516	3.3
75	α-Farnesene	502-61-4	Alkenes	93	93, 107, 69	28.63	1508	1519	N.F.
76	(−)-Calamenene	483-77-2	Alkenes	159	159, 202, 160	29.12	1523	1510	N.F.
77	δ-Cadinene	483-76-1	Alkenes	161	161, 134, 119	29.16	1524	1511	N.F.
78	Cubenene	29837-12-5	Alkenes	119	119, 161, 105	29.47	1532	1520	N.F.
79	α-Cadinene	24406-05-1	Alkenes	105	105, 161, 91	29.67	1538	1525	N.F.
80	α-Calacorene	21391-99-1	Alkenes	157	157, 142, 141	29.87	1542	1531	N.F.
81	E-Nerolidol	40716-66-3	Alcohols	69	69, 93, 41	30.76	1564	1555	250
82	*cis*-3-Hexenyl benzoate	25152-85-6	Esters	105	105, 82, 67	30.97	1570	1559	500
83	*n*-Hexyl benzoate	6789-88-4	Esters	123	123, 105, 77	31.25	1580	1567	73
84	*cis*-3-Hexenyl *n*-octanoate	61444-41-5	Esters	82	82, 67, 57	31.35	1562	1569	N.F.
85	Cedrol	77-53-2	Alcohols	150	150, 119, 95	32.02	1598	1586	0.5
86	Hexadecane	544-76-3	Alkanes	57	57, 71, 43	32.14	1600	1589	13,000,000
87	α-Corocalene	20129-39-9	Alkenes	185	185, 200, 143	32.95	1623	1610	N.F.
88	Di-epi-1,10-cubenol	73365-77-2	Alcohols	119	119, 204, 161	33.16	1614	1616	N.F.
89	α-epi-Cadinol	5937-11-1	Alcohols	161	161, 121, 95	33.74	1640	1631	N.F.
90	δ-Cadinol	19435-97-3	Alcohols	161	161, 119, 105	33.95	1645	1636	N.F.
91	α-Cadinol	481-34-5	Alcohols	121	121, 95, 79	34.28	1653	1643	N.F.
92	Cadalene	183-78-3	Alkenes	183	183, 198, 168	35.08	1674	1665	N.F.
93	Heptadecane	629-78-7	Alkanes	57	57, 71, 43	36.08	1700	1690	N.F.

^a^ CAS Registry Number is an abbreviation for Chemical Abstracts Service Registry Number. ^b^ Rt: retention time of volatiles on the DB-5ms column. ^c^ RI-1: data were taken from the literature (http://webbook.nist.gov/chemistry/ accessed on 20 January 2026). ^d^ RI-2: the retention index (RI)was computed using *n*-alkanes (C8–C25) under the same chromatographic conditions as the detected volatile compounds. ^e^ OTs in water, μg/kg. The values were based on previous studies [6,20]. N.F. means not found.

**Table 2:** Contents of volatiles in XYMJ green tea produced in different seasons (μg/kg, on dry weight).

No.	Name		Contents	
		Spring XYMJ	Summer XYMJ	Autumn XYMJ
	***Alcohols***			
1	*trans*-3-Hexenol	13.29 ± 0.38 ^a^	13.72 ± 0.83 ^a^	10.46 ± 3.14 ^a^
2	2-Heptanol	6.53 ± 0.03 ^a^	6.59 ± 0.46 ^a^	5.25 ± 0.7 ^b^
3	Benzyl alcohol	5.01 ± 0.17 ^a^	4.42 ± 0.49 ^a^	3.92 ± 0.82 ^a^
4	α-Phenylethanol	0.45 ± 0.12 ^a^	0.42 ± 0.32 ^a^	0.42 ± 0.1 ^a^
5	*cis*-Linalool oxide (furanoid)	5.59 ± 0.19 ^a^	5.3 ± 0.32 ^a^	5.58 ± 0.73 ^a^
6	*trans*-Linalool oxide (furanoid)	8.66 ± 0.44 ^a^	9.19 ± 0.6 ^a^	7.73 ± 0.89 ^a^
7	Linalool	110.45 ± 0.17 ^a^	124.18 ± 5 ^b^	89.03 ± 7.25 ^c^
8	Phenethyl alcohol	16.68 ± 0.45 ^a^	12.82 ± 1.66 ^b^	8.81 ± 1.3 ^c^
9	1-Nonanol	2.52 ± 0.11 ^a^	2.14 ± 0.1 ^b^	1.48 ± 0.09 ^c^
10	*trans*-Linalool oxide (pyranoid)	8.32 ± 0.09 ^a^	7.24 ± 1.03 ^ab^	6.07 ± 0.73 ^b^
11	Nerol	1.52 ± 0 ^a^	1.52 ± 0.14 ^a^	1.11 ± 0.17 ^b^
12	Geraniol	132.54 ± 2.27 ^a^	111 ± 7.58 ^b^	43.61 ± 2.95 ^c^
13	(*E*)-Nerolidol	8.01 ± 0.04 ^a^	6.86 ± 0.62 ^b^	6.01 ± 0.24 ^a^
14	Cedrol	N.D.	0.6 ± 0.05	N.D.
15	Di-epi-1,10-cubenol	7.7 ± 0.07 ^a^	4.82 ± 0.36 ^b^	4.07 ± 0.37 ^c^
16	α-epi-Cadinol	13.06 ± 0.02 ^a^	7.65 ± 0.6 ^b^	6.76 ± 0.59 ^b^
17	δ-Cadinol	2.14 ± 0.06 ^a^	1.32 ± 0.1 ^b^	1.15 ± 0.13 ^b^
18	α-Cadinol	1.59 ± 0.14 ^a^	1 ± 0.13 ^b^	0.93 ± 0.12 ^b^
	*subtotal*	344.06 ± 1.97 ^a^	320.77 ± 17.74 ^a^	202.42 ± 19.68 ^b^
	***Aldehydes***			
19	Hexanal	99.3 ± 1.18 ^a^	58.34 ± 4.86 ^b^	44.45 ± 7.12 ^c^
20	Heptanal	66.67 ± 1.32 ^a^	67.2 ± 5.95 ^a^	43.27 ± 5.47 ^b^
21	Benzaldehyde	15.2 ± 0.43 ^a^	15.01 ± 1.24 ^a^	17.12 ± 1.23 ^a^
22	Benzeneacetaldehyde	2.86 ± 0.21 ^a^	3.12 ± 0.63 ^a^	2.39 ± 1.19 ^a^
23	Nonanal	45.5 ± 2.36 ^a^	40.6 ± 3.78 ^a^	24.16 ± 3.38
24	Safranal	2.91 ± 0.11 ^b^	2.31 ± 0.09 ^c^	4.72 ± 0.4 ^a^
25	Decanal	2.19 ± 0.1 ^a^	1.81 ± 0.11 ^b^	1.22 ± 0.22 ^b^
26	β-Cyclocitral	7.58 ± 0.21 ^a^	8.16 ± 0.27 ^a^	8.61 ± 0.95 ^a^
27	β-Homocyclocitral	2.55 ± 0.07 ^a^	2.87 ± 0.04 ^a^	2.89 ± 0.28 ^a^
28	Citral	4.51 ± 0.16 ^a^	4.55 ± 0.4 ^a^	2.43 ± 0.26 ^b^
29	(*2E*,*6Z*)-Nonadienal	0.66 ± 0.09 ^a^	0.71 ± 0.08 ^a^	0.31 ± 0.02 ^b^
	*subtotal*	249.95 ± 3.27 ^a^	203.96 ± 16 ^b^	151.26 ± 15.7 ^c^
	***Alkenes***			
30	β-Myrcene	41.17 ± 0.42 ^a^	45.84 ± 0.88 ^ab^	61.32 ± 13.67 ^a^
31	Limonene	11.49 ± 0.03 ^b^	12.12 ± 0.24 ^b^	24.24 ± 6.82 ^a^
32	*cis*-β-Ocimene	24.42 ± 0.73 ^b^	25.49 ± 1.05 ^b^	43.7 ± 7.75 ^a^
33	p-Cymenene	4.97 ± 0.2 ^b^	4.28 ± 0.17 ^b^	8.94 ± 0.84 ^a^
34	(*E*)-4,8-Dimethylnona-1,3,7-triene	51.03 ± 2.14 ^a^	40.65 ± 0.63 ^a^	61.74 ± 20.73 ^a^
35	Allo-Ocimene	7.91 ± 0.2 ^b^	7.98 ± 0.24 ^b^	15.36 ± 3.47 ^a^
36	α-Cubebene	12.87 ± 0.26 ^a^	10.5 ± 0.52 ^a^	10.1 ± 1.92 ^a^
37	Copaene	3.08 ± 0.2 ^a^	2.38 ± 0.14 ^a^	3.96 ± 1.92 ^a^
38	Ylangene	2.81 ± 0.23 ^a^	2.15 ± 0.1 ^a^	3.6 ± 1.84 ^a^
39	β-Cubebene	5.8 ± 0.09 ^a^	4.41 ± 0.26 ^a^	2.91 ± 0.22 ^b^
40	(−)-β-Elemene	3.22 ± 0.15 ^a^	2.28 ± 0.46 ^a^	2.23 ± 0.54 ^a^
41	α-Gurjunene	0.96 ± 0.06 ^a^	0.74 ± 0.05 ^a^	1.14 ± 0.53 ^a^
42	Caryophyllene	6.26 ± 0.37 ^a^	3.97 ± 0.09 ^a^	5.87 ± 2.26 ^a^
43	γ-Elemene	4.67 ± 0.18 ^a^	1.2 ± 0.06 ^b^	1.51 ± 0.32 ^b^
44	Dihydrocurcumene	4.76 ± 0.23 ^a^	3.78 ± 0.3 ^a^	4.51 ± 1.01 ^a^
45	α-Humulene	6.13 ± 0.26 ^a^	5.38 ± 0.17 ^a^	7.66 ± 2.11 ^a^
46	(*E*)-β-Farnesene	2.12 ± 0.15 ^a^	1.26 ± 0.13 ^a^	1.24 ± 0.27 ^a^
47	Cadina-3,5-diene	4.33 ± 0.12 ^a^	3.45 ± 0.31 ^a^	4.26 ± 1.21 ^a^
48	*cis*-Muurola-4(15),5-diene	1.88 ± 0.07 ^a^	1.23 ± 0.03 ^a^	1.82 ± 0.75 ^a^
49	Cadina-1(6),4-diene	4.36 ± 0.32 ^a^	3.16 ± 0.24 ^a^	4.42 ± 1.7 ^a^
50	Bicyclosesquiphellandrene	5.85 ± 0.12 ^a^	4.47 ± 0.2 ^a^	5.08 ± 1.27 ^a^
51	Valencene	1.84 ± 0.07 ^a^	1.1 ± 0.15 ^ab^	0.98 ± 0.28 ^b^
52	α-Muurolene	4.36 ± 0.25 ^a^	2.95 ± 0.15 ^a^	4.09 ± 1.39 ^a^
53	α-Farnesene	0.88 ± 0.01 ^a^	0.7 ± 0.08 ^a^	0.82 ± 0.22 ^a^
54	(−)-Calamenene	68.39 ± 3.95 ^a^	47.77 ± 3.1 ^a^	59.88 ± 15.5 ^a^
55	δ-Cadinene	44.31 ± 2.73 ^a^	30.25 ± 1.59 ^a^	36.67 ± 11.78 ^a^
56	Cubenene	4.63 ± 0.18 ^a^	3.24 ± 0.24 ^a^	3.34 ± 0.85 ^a^
57	α-Cadinene	0.69 ± 0.01 ^a^	0.42 ± 0.01 ^a^	0.57 ± 0.23 ^a^
58	α-Calacorene	10.13 ± 0.72 ^a^	5.42 ± 0.46 ^a^	7.59 ± 1.77 ^a^
59	α-Corocalene	1.03 ± 0.03 ^a^	0.64 ± 0.07 ^a^	0.87 ± 0.22 ^a^
60	Cadalene	2.69 ± 0.19 ^a^	1.56 ± 0.2 ^a^	1.64 ± 0.28 ^a^
	*subtotal*	349.02 ± 14.31 ^a^	281.48 ± 9.4 ^a^	392.37 ± 102.54 ^a^
	***Esters***			
61	*trans*-3-Hexenyl butyrate	110.92 ± 12.18 ^a^	111.85 ± 3.69 ^a^	89.48 ± 3.66 ^b^
62	Methyl salicylate	36.4 ± 5.39 ^a^	38.4 ± 3.37 ^a^	24.11 ± 1.21 ^b^
63	*cis*-3-Hexenyl-α-methylbutyrate	58.16 ± 7.46 ^a^	61.41 ± 0.92 ^a^	50.54 ± 5.6 ^a^
64	*cis*-3-Hexenyl isovalerate	9.3 ± 0.93 ^a^	10.47 ± 0.34 ^a^	8.39 ± 1.13 ^a^
65	Vinyl hexanoate	3.53 ± 0.72 ^b^	4.42 ± 0.71 ^b^	6.87 ± 0.92 ^a^
66	Methyl *trans*-geranoate	3.71 ± 0.59 ^ab^	4.46 ± 0.31 ^a^	3.18 ± 0.39 ^b^
67	*cis*-3-Hexenyltiglate	7.74 ± 1.15 ^a^	5.33 ± 0.37 ^b^	3.49 ± 0.24 ^c^
68	*cis*-3-Hexenyl hexanoate	231.59 ± 27.79 ^b^	314.37 ± 11.84 ^a^	253.34 ± 13 ^b^
69	*n*-Hexyl hexanoate	14.32 ± 2.29 ^a^	16.94 ± 0.95 ^a^	9.13 ± 0.42 ^b^
70	*trans*-2-Hexenyl hexanoate	18.52 ± 2.62 ^a^	19.44 ± 1.12 ^a^	10.94 ± 0.75 ^b^
71	*cis*-3-Hexenyl *n*-octanoate	4.94 ± 0.88 ^a^	5.67 ± 0.52 ^a^	4.21 ± 0.12 ^a^
72	*cis*-3-Hexenyl benzoate	4.91 ± 0.75 ^a^	4.38 ± 0.44 ^ab^	3.38 ± 0.22 ^b^
73	*n*-Hexyl benzoate	0.8 ± 0.12 ^a^	0.71 ± 0.08 ^a^	0.61 ± 0.03 ^a^
	*subtotal*	548.53 ± 10.36 ^b^	597.85 ± 23.93 ^a^	467.69 ± 11.19 ^b^
	***Ketones***			
74	6-Methyl-5-hepten-2-one	7.6 ± 0.25 ^b^	8.93 ± 0.55 ^b^	11.5 ± 1.1 ^a^
75	Acetophenone	2.11 ± 0.05 ^a^	2.12 ± 0.19 ^a^	2.27 ± 0.19 ^a^
76	*cis*-Jasmone	26.9 ± 0.17 ^a^	13.93 ± 1.68 ^b^	8.76 ± 0.43 ^c^
77	Hexahydropseudoionone	0.57 ± 0.03 ^a^	0.49 ± 0.05 ^a^	0.56 ± 0.04 ^a^
78	α-Ionone	1.59 ± 0.02 ^a^	1.33 ± 0.05 ^a^	1.49 ± 0.25 ^a^
79	*trans*-Geranylacetone	2 ± 0.13 ^a^	2.16 ± 0.15 ^a^	1.97 ± 0.2 ^a^
80	Dehydro-β-ionone	N.D.	N.D.	0.68 ± 0.11
81	*trans*-β-Ionone	15.68 ± 0.3 ^a^	15.11 ± 0.97 ^a^	13.55 ± 1.38 ^a^
	*subtotal*	56.44 ± 0.51 ^a^	44.07 ± 3.2 ^b^	40.78 ± 3.49 ^b^
	***Alkanes***			
82	Theaspirane	5.97 ± 0.09 ^b^	4.72 ± 0.06 ^b^	9.24 ± 0.92 ^a^
83	Tetradecane	0.64 ± 0.08 ^a^	0.77 ± 0.02 ^b^	1.17 ± 0.16 ^a^
84	Pentadecane	1.47 ± 0.1 ^b^	1.64 ± 0.08 ^b^	2.08 ± 0.13 ^a^
85	Hexadecane	2.37 ± 0.15 ^a^	2.26 ± 0.06 ^a^	1.67 ± 0.24 ^a^
86	Heptadecane	0.86 ± 0.06 ^a^	0.75 ± 0.07 ^a^	N.D.
	*subtotal*	11.31 ± 0.3 ^b^	10.15 ± 0.14 ^b^	14.17 ± 0.94 ^a^
	***Aromatics***			
87	Styrene	25.95 ± 2.12 ^c^	41.51 ± 1.16 ^a^	32.38 ± 2.71 ^b^
88	Naphthalene	11.81 ± 0.38 ^b^	10.15 ± 1.25 ^b^	14.87 ± 1.27 ^a^
89	2-Methylnaphthalene	0.79 ± 0.04 ^a^	0.59 ± 0.11 ^ab^	0.45 ± 0.05 ^b^
90	Dehydro-ar-ionene	3.25 ± 0.17 ^b^	4.48 ± 0.16 ^b^	10.61 ± 1.27 ^a^
	*subtotal*	41.8 ± 1.53 ^b^	56.73 ± 1.87 ^a^	58.32 ± 3.24 ^a^
	***Others***			
91	2-Pentylfuran	84.23 ± 3.81 ^a^	34.28 ± 2.18 ^b^	36.39 ± 5.05 ^b^
92	Indole	29.96 ± 0.02 ^a^	15.81 ± 2.35 ^b^	13.04 ± 0.45 ^b^
93	Dibenzofuran	1.39 ± 0.03 ^b^	1.64 ± 0.14 ^ab^	1.78 ± 0.19 ^a^
	*subtotal*	115.58 ± 3.76 ^a^	51.72 ± 4.58 ^b^	51.21 ± 5.25 ^b^
	**Total**	1716.68 ± 28.41 ^a^	1566.72 ± 72.1 ^a^	1378.21 ± 124.63 ^b^

Data are presented as mean ± standard deviation (*n* = 3). Different superscript letters (a, b, c) within the same row indicate statistically significant differences (*p* < 0.05) as determined by one-way ANOVA followed by Tukey’s HSD post hoc test; N.D.: not detected.

**Table 3:** The volatiles with odor activity values (OAVs) in XYMJ.

No.	Volatiles	Odor Quality		OAV	
			Spring	Summer	Autumn
1	Linalool	citrus, floral, sweet	1546	1874	286
2	Nonanal	floral	124	122	14
3	Heptanal	green	72	79	10
4	Geraniol	sweet, floral	61	55	4
5	*cis*-3-Hexenyl hexanoate	fruity, green	48	65	10
6	*cis*-Jasmone	woody, floral	11	6	<1
7	β-Myrcene	citrus	8	10	2
8	Methyl salicylate	sweet	3	3	<1
9	Indole	floral	2	1	<1
10	Styrene	sweet	1	2	<1
11	*trans*-3-Hexenyl butyrate	green, sweet	<0.1	<0.1	<1

## Data Availability

Data is contained within the article or Supplementary Material.

## Supplementary Materials

The following supporting information can be downloaded at: https://www.mdpi.com/article/10.3390/biology15120925/s1, Table S1. Percentage contribution (%) of volatile chemical classes in Xinyang Maojian green tea across three seasons.
